# Risk factors of early bacterial infection and analysis of bacterial composition, distribution and drug susceptibility after cadaveric liver transplantation

**DOI:** 10.1186/s12941-023-00616-9

**Published:** 2023-07-31

**Authors:** Min Liu, Cuiying Li, Jing Liu, Qiquan Wan

**Affiliations:** 1 Department of Transplant Surgery, the Third Xiangya Hospital, Central South University, 410013 Changsha, China; 2 Engineering and Technology Research Center for Transplantation Medicine of National Health Commission, the Third Xiangya Hospital, Central South University, 410013 Changsha, China

**Keywords:** Liver transplantation, Bacterial infection, Drug resistance, Risk factor

## Abstract

**Background:**

This study provided a theoretical basis for the clinical diagnosis and treatment of bacterial infection after liver transplantation through analyzing the pathogenic distribution, drug sensitivity and risk factors of bacterial infection after liver transplantation.

**Methods:**

We collected clinical data from 207 recipients undergoing liver transplantation of graft from donation after citizens’ death donors in the Third Xiangya Hospital of Central South University from January 2019 to December 2021 and analyzed the composition and distribution of bacterial pathogens, drug resistance and risk factors of infection.

**Results:**

A total of 90 bacterial infections occurred in 55 recipients within two months after liver transplantation, and the incidence of bacterial infection was 26.6% (55/207). The gram-negative bacteria (46/90, 51.1%) were more prevalent than gram-positive bacteria (44/90, 48.9%). Common sites of infection were the abdominal/biliary tract (26/90, 28.9%), lung (22/90, 22.4%) and urinary tract (22/90, 22.4%). Fourteen cases (6.8%) died after liver transplantation. *Klebsiella pneumoniae* (17/90, 18.9%) was the most frequent gram-negative bacteria causing infection in liver transplant recipients and 58.7%, 50%, 80.4% and 89.1% of gram-negative bacteria were sensitive to amikacin, minocycline, tigecycline and polymyxin B, respectively. The most common gram-positive bacteria was *Enterococcus faecium* (30/90, 33.3%) and 97.7%, 100%, 86.4%, 100% and 100% of gram-positive bacteria were sensitive to vancomycin, teicoplanin, daptomycin, tigecycline and linezolid, respectively. Univariate analysis revealed that bacterial infection was associated with female, age (≥ 50 years old), preoperative albumin (≤ 30 g/L), operation duration (≥ 400 min), intraoperative blood loss (≥ 3000 ml) and postoperative ventilator support. Binary Logistic regression analysis showed that female (OR = 3.149, 95% CI: 1.418–6.993, *P* = 0.005), operation duration (≥ 400 min) (OR = 2.393, 95% CI: 1.202–4.765, *P* = 0.013) and intraoperative blood loss (≥ 3000 ml) (OR = 2.052, 95% CI: 1.007–4.183, *P* = 0.048) were independent risk factors for bacterial infection after liver transplantation.

**Conclusion:**

The incidence of early bacterial infection after liver transplantation was high, and the infection sites were mainly abdominal/biliary tract, respiratory tract and urinary tract. The most common pathogenic bacterium was gram-negative bacterium. Our study also identified several independent risk factors for bacterial infection after liver transplantation, including female gender, operation duration of 400 min or more, and intraoperative blood loss of 3000 ml or more. By addressing these risk factors, such as implementing strategies to optimize surgical procedures and minimize blood loss, healthcare professionals can work towards reducing the incidence of bacterial infections following liver transplantation.

## Background

With advancements in liver transplantation (LT) technology, many individuals afflicted with end-stage liver disease now have the opportunity to be reborn [1]. However, LT entails certain distinctive aspects that warrant consideration: [1] most patients who are malnutrition and immunocompromised have multiple organ function impairments before LT, which are leading to a high incidence of preoperative infection; [2] LT operation is intricate, lengthy, and traumatic, and often requires a large amount of intraoperative blood transfusion and infusion, thereby increasing the risk of fluid imbalances; [3] Invasive operations such as deep vein catheterization, indwelling urethral catheterization and tracheal intubation, coupled with broad-spectrum antibiotics, hormones and immunosuppressants, severely compromise patients’ immune systems [2, 3]. Consequently, the incidence of infections among LT recipients during the early postoperative period is significantly higher compared to recipients of other solid organ transplants. Such infections detrimentally impact the quality of life for LT recipients and, in severe cases, can lead to mortality. Research has indicated that bacterial infections, representing common and grave complications, predominantly occur within the first two months post-LT as a leading cause of death among LT recipients [4–7]. In addition, owing to the change in the immune status of transplant recipients, the spectrum of pathogenic bacteria manifests in a complicated and diversified trend. The emergence and rapid spread of multi-drug-resistant bacteria have become a global focus [7, 8]. Therefore, it is crucial to investigate the bacterial epidemiology and analyze the results of drug susceptibility after LT in our center for guiding clinical diagnosis and treatment, and ultimately improving the prognosis of LT recipients. Previous studies have predominantly focused on a single site of infection or a single pathogen, with a limited examination of the overall bacterial profile. In our present study, we collected clinical data of recipients following LT in our center and analyzed the bacterial pathogens, drug resistance and potential risk factors for the infection to provide a reference for clinical diagnosis and treatment.

## Methods

### Patients

The study was a retrospective case-control investigation, wherein we examined 207 recipients who received LT of graft from donation after citizens’ death donors in our hospital from January 2019 to December 2021. The collection of data encompassed clinical information, laboratory results and microbiology data. There were 172 males and 35 females. The range of ages was 19 to 68 years (46.0 ± 9.8). The cohort consisted of individuals with various underlying diseases, including hepatitis B cirrhosis/liver failure/liver cancer (n = 156), hepatitis C/E cirrhosis (n = 5), alcoholic cirrhosis (n = 14), mixed liver disease (n = 9), autoimmune hepatitis (n = 4), liver failure after LT (n = 4), cryptogenic cirrhosis (n = 4), Brugada syndrome (n = 4), Wilson disease (n = 3), primary biliary cirrhosis(n = 2), hepatic veno-occlusive disease (n = 1) and drug-induced liver injury (n = 1). The written informed consent was obtained from all the participants.

### Inclusion and exclusion criteria

We applied certain exclusion criteria, excluding individuals under the age of 18, those lacking essential clinical data, recipients with infections occurring within two weeks prior to LT, and cases of bacterial infection originating from the donor. Additionally, recipients who experienced perioperative mortality due to factors such as anesthesia accidents and surgical complications were excluded. Out of the initial 214 adult recipients who underwent LT, a total of 7 recipients were excluded from our study. Among them, 4 recipients had incomplete clinical data, one recipient tragically succumbed to massive hemorrhage during the operation, and 2 recipients were excluded due to bloodstream infections caused by donor-derived *Acinetobacter baumannii* and *Klebsiella pneumoniae*, respectively.

### Treatment methods

All recipients underwent modified piggyback LT under general anesthesia with tracheal intubation. We routinely performed cholecystectomy of the donor liver and placed the right subphrenic drainage tube and Venn’s foramina drainage tube. The left subphrenic drainage tube was placed in some recipients, and a “T” tube was placed in very few recipients for biliary drainage. Following surgery, each recipient received prophylactic third-generation cephalosporins or carbapenem antibiotics after surgery, based on pre and post-transplantation culture results and the antibiotics used prior to the surgery. The treatment course of antibiotics ranged from 3 to 6 days. Most recipients were immunologically induced with basiliximab. Posttransplant immunosuppressive therapy was maintained with tacrolimus and glucocorticoid. The trough concentration of tacrolimus was 8–10 µg/L in the first month and 6–8 µg/L in the second month after LT. Glucocorticoid was initially administered with intravenous methylprednisolone, which was transitioned to oral prednisone tablets around the 8th day. Meanwhile, recipients received mycophenolate mofetil or mycophenolate sodium enteric-coated tablets or rabbit anti-human thymocyte immunoglobulin as needed. The dosage of immunosuppressants was adjusted individually according to the presence of infection and rejection, and human immunoglobulin was used if necessary. All recipients were closely monitored in the intensive care unit of our transplant center recently after LT and we strictly implemented infection prevention and control measures for tracheal catheter, urinary catheter and deep vein catheterization after operation. Daily assessments were carried out, and catheters were promptly removed when appropriate. If there were no special circumstances, the tracheal cannulas were removed immediately after the recipient woke up. The urinary catheters were generally pulled out on the 3rd day and the central venous catheters were typically removed around the 7th day after LT.

### Microorganisms and culture methods

Cultures were obtained from various sources, including blood, urine, bile, wound drainage fluid, sputum, or bronchoalveolar lavage fluid. The intravenous blood collection for blood culture was under aseptic operation at two sites at the same time point. Eight to 10 ml of blood samples were injected into anaerobic and anaerobic culture bottles. Blood culture for aerobic and anaerobic bacteria was routinely performed once a day for 5 days after LT. For other cultures, specimens were obtained for routine bacterial culture once a day for 3–7 days after LT if the corresponding specimen can be collected. Blood samples and other specimens were immediately sent to the microbiology laboratory for bacterial culture. Blood samples were cultured and monitored using the BD9120 automatic blood culture instrument (Becton Dickinson, USA). All bacteria were identified by Bruker mass spectrometer. The CDC/NHSN criteria were used to determine bacteremia and other infections [9]. The source of infection was defined as a culture-positive site of infection accompanied by clinical signs of active infection (e.g., chills, fever, hypotension or by imaging such as CT or chest X-ray) [9]. The isolation of a bacterium other than normal skin flora (Diphtheroids, Bacillus spp., or Coagulase-negative Staphylococcus) in one culture with signs of infection or the isolation of a bacterium from at least two consecutive cultures correlated with signs of infection. All intermediate conditions were categorized as drug resistance during drug susceptibility analysis.

### Information content and access method

All of the LT recipients were followed up for 2 months. The relevant data, including the preoperative, intraoperative and postoperative general conditions of the recipients, and all possible demographic, laboratory and clinical data related to the development of infection, as well as postoperative survival, were obtained by consulting electronic medical records, outpatient and telephone follow-up.

### Data analyses and statistics

We used SPSS 22.0 software to analyze the data. Continuity variables were expressed as mean ± standard deviation or median (interquartile range [IQR]), and classification data were expressed as percentages. Univariate analysis was performed using the Chi-square test, continuous correction, or Fisher’s exact test. We included the variables with statistical significance in the univariate analysis for the final binary logistic regression model. Odds ratio (OR) values and 95% confidence intervals (95%CI) were used to describe independent factors associated with bacterial infection. The difference was considered statistically significant when the *P* value was less than 0.05 in the two-tail test.

## Results

### The basic characteristics and prognosis of the LT recipients

A total of 207 LT recipients were included in our study from January 2019 to December 2021. The demographic data, laboratory indicators and clinical data of the LT recipients were presented in Table 1. 172 (83.1%) of 207 recipients were males with an average age of 46 (19–69) years. The median length of hospital stay was 8 [1–24] days before LT and 26 [22–30] days after LT. The median score of end-stage liver disease (MELD) was 25 [16–30]. Sixty-eight (32.8%) recipients received antibiotics within 15 days before LT. Ninety-nine recipients (47.8%) developed an infection within 2 months before LT, including 73 recipients (35.2%) with pulmonary infection and 18 recipients with multiple site infection (all of these 18 recipients had pulmonary infection). The primary liver diseases among the recipients were hepatitis B cirrhosis/fulminant liver failure/hepatocellular carcinoma and alcoholic cirrhosis, accounting for 156 recipients (75.4%) and 14 recipients (6.8%), respectively. There were 9 recipients with multiple etiologies of liver disease, including 7 recipients with hepatitis B virus infection. There were 27 recipients (13%) with type 2 diabetes before LT. One hundred and forty-four (69.6%) recipients were immunologically induced with basiliximab. Among 144 recipients (69.6%), 1 recipient (0.5%) received 60 mg, 108 recipients (52.2%) received 40 mg and 35 recipients (16.9%) received 20 mg basiliximab. There were 63 recipients (30.4%) with no basiliximab. Tacrolimus was used in 206 recipients (99.5%) after LT, and cyclosporin A was used in 1 recipient with type 2 diabetes (0.5%) due to poor postoperative glycemic control. One hundred and twenty-five recipients (60.4%) received mycophenolate mofetil, 46 recipients (22.2%) received mycophenolate sodium enteric-coated tablets, and no recipients received rapamycin. Twenty-six recipients (12.6%) developed acute rejection. Fourteen recipients died within 2 months after LT. One recipient died of severe pneumonia and multiple organ dysfunction caused by *Aspergillus fumigatus* and *A. baumannii* the 27th day after LT. One recipient died of severe pneumonia and multiple organ dysfunction caused by *K. pneumoniae* on the second day after LT. One recipient died of severe pneumonia and multiple organ dysfunction caused by *Pneumocystis jirovecii* on the 11th day after LT. Two recipients died of septic shock and multiple organ failure caused by *Enterococcus faecium*. Two recipients died of unexplained intracranial hemorrhage. One recipient died of hemorrhagic shock due to esophageal ulcerative bleeding on the 29th day after LT. Two recipients died of graft liver failure caused by severe rejection. One recipient died of asphyxia caused by intensive care unit-acquired weakness. One recipient died of hemorrhagic shock caused by extensive bleeding of gastric mucosa on the 6th day after LT. One recipient died of abdominal hemorrhagic shock and multiple organ failure. One recipient died of cerebral abscess combined with cerebral hernia on the 30th day after LT. There were 10 recipients with bacterial infection among 14 deceased LT recipients.

**Table 1:** Demographic, laboratory, and clinical variables of 207 LT recipients

Characteristics	Value
Age (year)	46.0 ± 9.8
Gender (%) Male Female	172(83.1%) 35(16.9%)
Length of hospital stay before LT (days), median (IQR)	8(1–24)
Preoperative MELD score, median (IQR)	25(16–30)
Antibacterial drug use within 15 days before LT (%)	68(32.8)
Infection within 2 months before LT (%)	99(47.8)
Pulmonary infection	73(35.2)
Abdominal/biliary tract infection	6(2.9)
Bloodstream infection	1(0.5)
Urinary tract infection	1(0.5)
Multisite infection	18(8.7)
Primary disease for LT (%)	
Hepatitis B cirrhosis, liver necrosis and liver cancer	156(75.4)
Hepatitis C or E cirrhosis	5(2.4)
Alcoholic cirrhosis	14(6.8)
Autoimmune hepatitis	4(1.9)
Others	19(9.2)
Mixed liver disease	9(4.3)
Preoperative type 2 diabetes (%)	27(13.0)
Preoperative WBC (×10^9^/L), median (IQR))	5.17(3.5–8.08)
Preoperative lymphocyte count (×10^9^/L), median (IQR)	0.82(0.51–1.24)
Preoperative platelet count (×10^9^/L), median (IQR)	65(43–96)
Preoperative albumin (g/L), median (IQR))	33.9(30.5–36.9)
Cold ischemia time of donor (hrs), median (IQR)	5.6(4.1–7.4)
Time of operation (min), median (IQR)	365(335–422)
The intraoperative blood loss (ml), median (IQR)	3000(2000–4200)
The intraoperative RBC transfusion (U), median (IQR)	12.5(9–17)
Postoperative ventilator support (%)	16(7.7)
Transplantation or open laparotomy again (%)	7(3.4)
Postoperative duration of indwelling urethral catheter (days), median (IQR)	3(3–5)
Postoperative methylprednisolone dosage (mg), median (IQR)	1570(1360–1760)
Immunosuppressant (%)	
Tacrolimus	206(99.5)
Cyclosporin A	1(0.5)
Mycophenolate mofetil	125(60.4)
Mycophenolate sodium enteric-coated tablets	46(22.2)
Glucocorticoids	207(100)
Antithymocyte globulin	14(6.8)
Basiliximab	144(69.6)
ALT on day 1 after surgery (U/L), median (IQR)	690(395–1185)
Serum creatinine on day 3 after surgery (mg/ml), median (IQR)	0.8(0.7–1.2)
Recipients with bacterial infection (%)	55(26.6)
Gram-negative infection	20(9.7)
Gram-positive infection	23(11.1)
Mixed infection	12(5.8)
Acute rejection (%)	26(12.6)
Deaths (%)	14(6.8)

ALT, alanine aminotransferase; IQR, interquartile range; LT, liver transplant; MELD, Model for End-Stage Liver Disease; RBC, red blood cell; WBC, white blood cell

### Classification of infecting pathogens, location and timing of infection

Fifty of the 207 recipients developed 90 strains of bacterial infection. Gram-negative bacteria were dominant in the composition of pathogenic bacteria (46/90, 51.1%). The common gram-negative bacteria were *K. pneumoniae* (17/90, 18.9%), *A. baumannii* (10/90, 11.1%), *Stenotrophomonas maltophilia* (5/90, 5.6%) and *Escherichia coli* (4/90, 4.4%). The most prevalent gram-positive bacteria was *E. faecium* (30/90, 33.3%), followed by *Enterococcus faecalis* (9/90, 10%) (Table 2). The sites of infection in 55 recipients were abdominal cavity/biliary tract (26/90, 28.9%), respiratory tract (22/90, 22.4%), urinary tract (22/90, 22.4%), bloodstream (19/90, 21.1%) and surgical incision (1/90, 1.1%). The most common site of infection by gram-negative bacteria was the respiratory tract. The most common site of infection by gram-positive bacteria was the urinary tract (Table 3).

**Table 2:** The composition of 90 bacterial pathogens in 55 LT recipients

Pathogen	The number of strains(90)	Percentage (%)
Gram-positive bacteria	44	48.9
*Enterococcus faecium*	30	33.3
*Enterococcus faecalis*	9	10
*Staphylococcus aureus*	3	3.3
*Staphylococcus haemolyticus*	1	1.1
*Enterococcus gallinarum*	1	1.1
Gram-negative bacteria	46	51.1
*Klebsiella pneumoniae*	17	18.9
*Acinetobacter baumannii*	10	11.1
*Stenotrophomonas maltophilia*	5	5.6
*Escherichia coli*	4	4.4
*Pseudomonas aeruginosa*	2	2.2
*Enterobacter cloacae*	1	1.1
*Enterobacter aerogenes*	1	1.1
*Salmonella enteritidis*	1	1.1
*Acinetobacter johnsonii*	1	1.1
*Burkholderia cepacia*	1	1.1
*Sphingomonas paucimobilis*	1	1.1
*Ralstonia pickettii*	1	1.1
*Shewanella putrefaciens*	1	1.1

**Table 3:** Source of specimens of pathogenic bacteria

	Respiratory tract	Abdominal cavity/ biliary tract	Urinary tract	Blood stream	Surgical incision
Gram-negative bacteria					
*Klebsiella pneumoniae* (17)	5	5	1	6	0
*Escherichia coli* (4)	0	1	3	0	0
Other Enterobacteriaceae (3)	0	0	1	2	0
*Acinetobacter baumannii* (10)	5	3	1	1	0
*Stenotrophomonas maltophilia* (5)	4	1	0	0	0
*Pseudomonas aeruginosa* (2)	2	0	0	0	0
Other non-fermenting bacteria (5)	3	1	0	1	0
Gram-positive bacteria					
*Enterococcus faecium* (30)	1	10	13	5	1
*Enterococcus faecalis* (9)	0	3	3	3	0
*Staphylococcus aureus* (3)	2	1	0	0	0
*Staphylococcus haemolyticus* (1)	0	0	0	1	0
*Enterococcus gallinarum* (1)	0	1	0	0	0
total (%)	22(24.4)	26(28.9)	22(24.4)	19(21.1)	1(1.1)

### Analysis of drug resistance in early bacterial infection

As shown in Table 4, the antibiotic resistance rate of gram-negative bacteria was high, including the third and fourth-generation cephalosporins, aztreonam, piperacillin, tazobactam, cefoperazone, sulbactam, imipenem, cilastatin, meropenem, levofloxacin, and sulfamethoxazole-trimethoprime. However, there were some antibiotics that showed relatively higher sensitivity against gram-negative bacteria, including minocycline (resistance rate 50%), amikacin (41.3%), tigecycline (19.6%) and polymyxin B (10.9%). Except for natural resistance of *Rolstonia pederi* and *Burkholderia onioniae* in polymyxin B, the resistance rate of other gram-negative bacteria to polymyxin B was only 6.8% (3/44). It was low for the resistance rate of *K. pneumoniae* to tigecycline (23.5%), amikacin (17.6%) and polymyxin B (5.9%). *A. baumannii* showed a low resistance rate to tigecycline (30%) and polymyxin B (0%). Most gram-positive bacteria had high resistance rates to clinically anti-gram-positive drugs with a resistance rate of more than 80%, such as penicillin G, levofloxacin, ampicillin and erythromycin (Table 5). It was 43.2% for the resistance rate of high-concentration gentamicin. Three strains of *Staphylococcus aureus* were all methicillin-resistant *S. aureus* (MRSA), and the resistance rate of *S. aureus* to erythromycin, levofloxacin, penicillin, ampicillin and high concentration of gentamicin was more than 50%. In *Enterococcus*, the resistance rate of *E. faecium* to most antibiotics was higher than that of *E. faecalis*. Interestingly, 6 strains of daptomycin-resistant or intermediate strains were detected in *E. faecalis*, warranting further investigation. The sensitivity rate of gram-positive bacteria to teicoplanin, tigecycline and linezolid reached 100%, and the resistance rate to vancomycin was only 2.3%. Except for the effect of one *Enterococcus gallinarum* on natural resistance to vancomycin, vancomycin was sensitive to all other gram-positive bacteria. Daptomycin was sensitive to all gram-positive bacteria except *E. faecalis*.

**Table 4:** Rate of drug-resistance of Gram-negative bacteria to 13 common used antibiotics (n, (%))

Antibiotics	Antimicrobial resistance rates(%)						
	*Klebsiella pneumoniae* (17)	*Escherichia coli*(4)	Other Enterobacteriaceae (3)	*Acinetobacter baumannii* (10)	*Stenotrophomonas maltophilia* (5)	Other non- fermenting bacteria (5)	Total resistant rate (45)
Ceftazidime	10(58.8)	3(75)	1(33.3)	8(80)	0(0)	3(42.9)	54.3
Cefepime	11(64.7)	3(75)	0(0)	9(90)	5(100)	4(57.1)	69.6
Piperacillin tazobactam	13(76.5)	1(25)	2(66.7)	9(90)	5(100)	5(71.4)	76.1
Cefoperazone sulbactam	10(58.8)	1(25)	2(66.6)	8(80)	0(0)	3(42.9)	52.2
Aztreonam	10(58.8)	2(50)	1(33.3)	7(70)	5(100)	4(57.1)	63
Imipenem	9(52.9)	1(25)	1(33.3)	8(80)	5(100)	5(71.4)	63
Meropenem	10(58.8)	1(25)	0(0)	8(80)	5(100)	5(71.4)	63
Amikacin	3(17.6)	0(0)	1(33.3)	8(80)	5(100)	2(28.6)	41.3
Levofloxacin	12(70.6)	3(75)	1(33.3)	9(90)	1(20)	1(14.3)	58.7
Cotrimoxazole	10(58.8)	1(25)	2(66.7)	8(80)	1(20)	5(71.4)	63
Minocycline	14(82.4)	1(25)	2(66.7)	4(40)	0(0)	2(28.6)	50
Tigecycline	4(23.5)	0(0)	0(0)	3(30)	0(0)	2(28.6)	19.6
Polymyxin B	1(5.9)	0(0)	0(0)	0(0)	0(0)	4(57.1)	10.9

**Table 5:** Rate of drug-resistance of Gram-positive cocci to 10 common used antibiotics (n, (%))

Antibiotics	Antimicrobial resistance rates(%)					
	*Enterococcus faecium* (30)	*Enterococcus faecalis* (9)	*Staphylococcus aureus* (3)	*Staphylococcus haemolyticus* (1)	*Enterococcus gallinarum* (1)	Total resistance rate (44)
Erythromycin	28(93.3)	8(88.9)	2(66.7)	1(100)	0(0)	88.6
Levofloxacin	28(93.3)	6(66.7)	2(66.7)	1(100)	0(0)	84.1
Penicillin	29(96.7)	4(44.4)	3(100)	1(100)	0(0)	84.1
Ampicillin	28(93.3)	5(55.6)	3(100)	1(100)	0(0)	84.1
High concentration of gentamicin	13(43.3)	3(33.3)	2(66.7)	1(100)	0(0)	43.2
Daptomycin	0(0)	6(66.7)	0(0)	0(0)	0(0)	13.6
Tigecycline	0(0)	0(0)	0(0)	0(0)	0(0)	0
Vancomycin	0(0)	0(0)	0(0)	0(0)	1(100)	2.3
Teicoplanin	0(0)	0(0)	0(0)	0(0)	0(0)	0
Linezolid	0(0)	0(0)	0(0)	0(0)	0(0)	0

### Analysis of risk factors for early bacterial infection

Univariate analysis showed that being female (*P* = 0.005), age ≥ 50 years old (*P* = 0.014), preoperative albumin ≤ 30 g/L (*P* = 0.041), operative duration ≥ 400 min (*P* = 0.004), intraoperative blood loss ≥ 3000 ml (*P* = 0.004), postoperative ventilator support (*P* = 0.027) were correlated with the occurrence of bacterial infection (Table 6).

Binary Logistic regression analysis identified that being female [OR = 3.149, 95%CI: 1.418–6.993, *P* = 0.005], operation duration ≥ 400 min [OR = 2.393, 95%CI: 1.202–4.765, *P* = 0.013] and intraoperative blood loss ≥ 3000 ml [OR = 2.052, 95%CI: 1.007–4.183, *P* = 0.048] were independent risk factors for bacterial infection after LT (Table 7).

**Table 6:** Univariate analysis of risk factors for infections due to bacteria in LT recipients

Variables	Infected recipients (55)	Uninfected recipients (152)	*P* value
Univariate analysis			
Female	16	19	0.005
Age ≥ 50 years	28	49	0.014
Preoperative MELD score ≥ 25	33	71	0.091
Length of hospital stay before LT ≥ 7 days	34	82	0.314
Antibiotic use within 15 days before LT	21	47	0.326
Hepatitis B cirrhosis/liver necrosis/liver cancer	40	116	0.597
Alcoholic cirrhosis	4	10	1
Preoperative type 2 diabetes	9	18	0.394
Infection within 2 months before LT	31	68	0.139
Preoperative WBC ≤ 4 × 10^9^/L	13	54	0.106
Preoperative lymphocyte count < 0.5 × 10^9^/L	9	42	0.097
Preoperative platelet count < 50 × 10^9^/L	16	54	0.387
Preoperative albumin ≤ 30 g/L	17	27	0.041
Cold ischemia time of donor > 360 min	25	70	0.939
Duration of operation ≥ 400 min	27	42	0.004
The intraoperative blood loss ≥ 3000ml	40	76	0.004
The intraoperative RBC transfusion ≥ 12U	37	82	0.087
Basiliximab ≥ 40 mg	32	77	0.338
Antithymocyte globulin	5	9	0.625
Methylprednisolone > 1500 mg	31	90	0.714
ALT on day 1 after LT > 1000U/L	22	41	0.2
Serum creatinine on day 3 after LT > 1.5 mg/dL	9	25	0.989
Postoperative ventilator support	8	8	0.027
Reoperation	3	4	0.577
Acute rejection	4	22	0.167

**Table 7:** Binary logistic regression analysis of risk factors for infections due to bacteria in LT recipients

Variables	B	S.E.	Waldχ^2^	*P* value	OR (95%CI)
Female	1.147	0.407	7.944	0.005	3.149(1.418–6.993)
Duration of operation ≥ 400 min	0.873	0.351	6.17	0.013	2.393(1.202–4.765)
Intraoperative blood loss ≥ 3000ml	0.719	0.363	3.915	0.048	2.052(1.007–4.183)

## Discussion

The complications of infection and rejection pose significant risks to the survival of LT recipients, despite the reported one-year survival rates of over 80% in most transplant centers [3, 4]. Infection has become the most important cause of hospital readmission and death of postoperative patients due to the rapid development of immunosuppressive therapy in recent years. The most common pathogen is bacteria [4, 10]. Some research showed that the incidence of bacterial infection was from 14 to 71.1% after LT [11, 12]. And the bacterial infection rate was 26.6% in our study, which was lower than the rates reported in two studies conducted in Zhejiang Province, China (68.6% [13] and 51.8% [14]). This difference may be attributed to variations in the definition of infection or the duration of postoperative follow-up. Hepatitis B-related liver diseases were still the main primary diseases for LT [14, 15], and the primary diseases in this study were mainly hepatitis B-related cirrhosis, liver necrosis and tumor. The mortality rate of LT recipients was 6.8% in our study, which was slightly lower than 8.2% reported by Jafarpour Z [5] and 8.1% reported by Zhang ML [14]. It is worth noting that 10 of 14 dead recipients had bacterial infections, and among them, 6 recipients died of severe infection, which was the main cause of death in our center. We should pay more attention to infection, as a relatively controllable factor, which has become the main cause of death compared with other causes.

Abdominal cavity and biliary tract have consistently been identified as the most common site of bacterial infection after LT [4, 5, 16]. The proportion of pulmonary, urinary tract, abdominal/biliary tract infections was similar in this study. Pulmonary infection accounted for 74.7% (74/99) of the preoperative infection cases in our center, and all the preoperative multiple-site infections included pulmonary infection, which might explain the high rate of postoperative pulmonary infection in this study.

In addition, the number of cases of urinary tract infection in our study was slightly lower than that of abdominal cavity/biliary tract, but it was higher than in other reports [17, 18]. Common risk factors for urinary tract infections were age, female sex, diabetes, urinary abnormalities, history of urinary infections, and long-term indwelling urethral catheters [19]. In this study, the proportion of preoperative urinary tract infection was not high (only 1 recipient), which did not match the high postoperative urinary tract infection rate. However, there are currently few studies on urethral catheter indwelling time after LT [4]. In fact, the median indwelling duration of the urethral catheter in this study was shorter than in other studies [20, 21], and the incidence of urinary tract infection within two days after surgery was higher in this study. The possibility of infection caused by catheterization could not be ruled out. At the same time, bloodstream infection caused by bacteria after LT accounted for a high proportion (21.1%), and urinary tract infection might exist as a part of systemic infection. However, the underlying causes of the high rate of postoperative urinary tract infections need to be further explored. Typically, catheter-related urinary tract infections occur when the catheter is in place for three days or longer. In this study, urinary system infections occurred earlier, which was also the reason why we did not include catheter indwelling duration as a risk factor for infection.

In this study, gram-negative bacteria were the common pathogens causing early post-transplant infection (51.1% of all bacterial infections), which is consistent with some previous studies [22, 23]. However, some studies have shown that gram-positive bacteria were the main pathogenic bacteria [6, 24, 25]. This difference in bacterial etiology might be the result from different infection prevention protocols for each center and the different geographical distribution of common pathogens. The common gram-negative bacteria were *K. pneumoniae* (18.9%), *A. Baumannii* (11.1%) and *S. maltophilia* (5.6%), which partially aligns with the report from Freire MP et al. [18]. The difference was that the incidence of *S. maltophilia* infection in this study was relatively high. It has occupied the third place in gram-negative bacterial infection after LT, which might be related to the extensive use of cephalosporins and carbapenem drugs which increased infections by opportunistic pathogens. *E. faecium* (33.3%) accounted for the largest proportion of gram-positive bacteria, which was the same as some studies [6, 25].

LT recipients had a high risk for multidrug-resistant bacterial infections due to multiple hospitalizations, invasive surgeries, and frequent use of antibiotics [26, 27]. At the same time, the infection progressed rapidly and LT recipients with an infection often had a poor prognosis because of a low immune function. Strong antibacterial drugs were often applied in large doses and for a long time, contributing to the problem of bacterial drug resistance. Most gram-negative bacteria in our center were resistant to the third and fourth generation of cephalosporins, aztreonam, piperacillin/tazobactam, cefoperazone/sulbactam, imipenem-cilastatin, meropenem, levofloxacin and sulfamethoxazole-trimethoprim, while they were sensitive to polymyxin B, tigecycline, amikacin and minocycline. Carbapenems were considered to be the first choice for the treatment of broad-spectrum β-lactamase-positive gram-negative bacteria, leading to a serious carbapenem resistance situation in the world. In our study, although the drug resistance rate was different from that proposed by Zhong L et al. [28], the isolated *Pseudomonas aeruginosa* and *A. baumannii* showed obvious resistance to carbapenems and *K. pneumoniae*, as the largest number of strains in the present study, also showed strong resistance to carbapenems. Meanwhile, only polymyxin B maintained high sensitivity to the above three bacteria. Most gram-positive bacteria were resistant to penicillin G, levofloxacin, ampicillin and erythromycin, and the resistance rate was close to 50% to high-concentration of gentamicin. MRSA and Vancomycin-resistant *Enterococcus* (VRE) still posed serious threats to LT recipients [24, 28]. Fortunately, no VRE was isolated except for *E. gallinarum*, which was naturally resistant for vancomycin, and the three strains of MRSA were sensitive to daptomycin, teicoplanin, vancomycin, tigecycline and linezolid. The low incidence of VRE in our center was related to the low use of vancomycin in our center for its renal toxicity. In general, the drug resistance rate of gram-positive bacteria was lower than that of Gram-negative bacteria, so the drug selectivity was much better. Absolutely, it is crucial to remain cautious regarding the increasing prevalence of multi-drug-resistant gram-positive bacteria. For recipients who were considered to have the possibility of postoperative bacterial infection clinically, antibiotics that could cover the most common and virulent pathogens should be applied as early as possible according to local bacterial epidemiology and drug susceptibility monitoring data, meanwhile medication should be adjusted in time according to drug susceptibility results and efficacy to avoid overuse of antibiotics and increase of drug resistance rate.

In some studies, bacterial infection was significantly associated with age, sex, MELD score, severe hepatitis, mechanical ventilation, length of stay after transplantation, renal failure, portal vein thrombosis, and complications of the biliary tract [13, 14, 28–32]. It was important to identify preventable risk factors to reduce the incidence of infection after LT. We examined the contribution of 25 risk factors for bacterial infection, including demographic, clinical, and microbiological variables and concluded that three factors were independently associated with a higher risk of infection. Our present study found that the duration of operation of more than 400 min was an independent risk factor for postoperative bacterial infection in LT. The association between operation time and complications has been demonstrated in many studies [33–35]. A multicenter study indicated that the risk of surgical complications increased with prolonged operation time, and the incidence of postoperative complications, including infection, increased if operation time was over two hours [36]. Some studies have found that most infections occurring within four weeks after LT were related to surgical techniques [13]. Some studies showed that operation time was an independent risk factor for early infection after LT [37–41]. The long procedure time might increase the chances of bacterial contamination in the operating field, reflecting the technical difficulty of the procedure. Therefore, reducing the operation time by improving the technique or adjusting the operation and anesthesia plan is the best way to reduce infection. In addition, our study also found that being female was an independent risk factor for bacterial infection after LT, which was consistent with some previous findings [4, 42, 43]. Currently, many studies have described gender differences in etiology, disease severity and outcome of LT recipients, which might be related to preoperative disease severity, postoperative renal function assessment differences, female body characteristics, gender mismatch between donors and recipients, and autoimmunity [44]. However, the specific reasons remain to be further studied. This present study also found that intraoperative blood loss of more than 3000 ml was an independent risk factor for bacterial infection after LT, which was consistent with the findings of Kaido T et al. [45]. And they found that massive blood loss during surgery was an independent risk factor for bacterial bloodstream infection after living donor LT [45].

The limitations of this present study are as follows: First, it is a retrospective study. Retrospective studies are inherently susceptible to missing data and potential biases. While efforts were made to collect relevant clinical data, there is a possibility of missing important information, such as the nutritional status of patients, anhepatic phase duration, and postoperative biliary leakage, which could have influenced the results and data analysis. Second, some studies have shown apparent differences in risk factors for different sites of infection. We did not distinguish the site of infection for risk factors analysis, which may have a certain impact on the results. In addition, bacterial colonization was not routinely monitored in this study, leading to a lack of preoperative donor and recipient microbial colonization data. Lastly, the study being conducted at a single center limits the generalizability of the findings.

We found a high rate of drug resistance to bacterial infection after LT in our center. While there are still available treatment options, the emergence of drug-resistant bacteria is concerning. Infections are significant preventable factors in LT morbidity and mortality, underscoring the need for attention and interventions targeting preventable risk factors in each center. By adapting interventions based on local factors and focusing on modifiable risk factors, centers can work towards reducing the incidence of bacterial infections after LT.

## Conclusions

The occurrence of early bacterial infection following liver transplantation was elevated, with the infection primarily affecting the abdominal/biliary tract, respiratory tract, and urinary tract. The predominant causative organism was gram-negative bacteria. Factors such as female gender, extended operative duration (≥ 400 min), and intraoperative blood loss (≥ 3000 milliliters) were identified as independent risk factors for bacterial infection subsequent to liver transplantation. Enhancing surgical techniques, minimizing operation duration, and mitigating intraoperative blood loss could potentially contribute positively to the reduction of bacterial infection after liver transplantation.

## Data Availability

The datasets generated during and/or analyzed during the current study are available from the corresponding author on reasonable request.

## Abbreviations

LT: Liver transplantation
IQR: Interquartile range
OR: Odds ratio
CI: Confidence intervals
MELD: Model for End-Stage Liver Disease
WBC: White blood cell
RBC: Red blood cell
ALT: Alanine aminotransferase
MRSA: Methicillin-resistant *S. aureus*
VRE: Vancomycin-resistant *Enterococcus*
